# Draft Genome Sequences of Six Lactobacillus gasseri and Three *Lactobacillus paragasseri* Strains Isolated from the Female Bladder

**DOI:** 10.1128/MRA.00973-19

**Published:** 2019-09-12

**Authors:** Catherine Putonti, Virina Akhnoukh, Zoe Anagnostopoulos, Maria Bilek, John Colgan, Samir El Idrissi, Karolina Magnuszewski, Steven Marino, Matthew Szymulewski, Megan Tyahla, Viktoriya Yanchuk, Taylor Miller-Ensminger, Mary Batrich, Bridget Brassil, Rita Mormando, Genevieve Johnson, Alan J. Wolfe

**Affiliations:** a Bioinformatics Program, Loyola University Chicago, Chicago, Illinois, USA; b Department of Biology, Loyola University Chicago, Chicago, Illinois, USA; c Department of Computer Science, Loyola University Chicago, Chicago, Illinois, USA; d Department of Microbiology and Immunology, Stritch School of Medicine, Loyola University Chicago, Maywood, Illinois, USA; e Department of Chemistry and Biochemistry, Loyola University Chicago, Chicago, Illinois, USA; f Niehoff School of Nursing, Stritch School of Medicine, Loyola University Chicago, Maywood, Illinois, USA; Georgia Institute of Technology

## Abstract

Lactobacilli are dominant members of the healthy female bladder microbiota. Here, we report the complete genome sequences of six Lactobacillus gasseri and three Lactobacillus paragasseri strains isolated from catheterized urine samples. These *L. paragasseri* genomes are the first publicly available sequences of the species from the bladder.

## ANNOUNCEMENT

Lactobacillus is a commensal bacterium in the human body and is a key component of the healthy urinary and vaginal microbiota (1). The family Lactobacillaceae has one of the highest rates of incidence compared to those of other bacterial families in the urinary tract (2). Lactobacillus gasseri is a predominant species in the human microbiota and is able to prevent other bacteria from growing in the same environment, protecting the host from pathogens (3). *Lactobacillus paragasseri* was classified as a novel species in 2018 (4) and, until now, has not been characterized in the urinary tract.

Catheterized urine samples were collected from women as part of prior institutional review board (IRB)-approved studies (5–9). Bacteria were isolated from these samples using the enhanced quantitative urine culture (EQUC) method (9) and stored at −80°C. We selected nine strains in our collection for whole-genome sequencing; these strains were identified as L. gasseri by matrix-assisted laser desorption ionization–time of flight mass spectrometry (MALDI-TOF) mass spectrometry. Freezer stocks for each of the nine strains were first streaked on Columbia colistin-nalidixic acid agar with 5% sheep blood plates (catalog number 221353; BD) and incubated at 35°C in 5% CO_2_ for 48 hours. A single colony was then selected and grown in MRS liquid medium at 35°C in 5% CO_2_ for 48 hours. DNA was extracted with the Qiagen DNeasy UltraClean microbial kit, and the DNA was quantified by a Qubit fluorometer. DNA libraries were constructed (Nextera XT library prep kit) and sequenced using the MiSeq reagent kit v2, producing 250-bp paired-end reads (minimum, 266,494 pairs; maximum, 1,342,972 pairs; average, 524,976 pairs). The raw reads were trimmed using Sickle v1.33 (https://github.com/najoshi/sickle) and then assembled with SPAdes v3.13.0 (10) (parameters, “only-assembler” option for k = 55, 77, 99, and 127). The assembled contigs were evaluated for genome completeness and contamination by CheckM v1.0.12 (11), and genome coverage was calculated using BBMap v38.47 (https://sourceforge.net/projects/bbmap/). Genome annotations were performed using PATRIC v3.5.43 (12) and the NCBI Prokaryotic Genome Annotation Pipeline (PGAP) v4.8 (13). The PGAP annotations are published with the deposited genome assemblies. A phylogenetic tree was derived with RAxML v8.2.11 in PATRIC using the PATRIC annotations and the codon tree method (12). Unless otherwise noted, default parameters were used for all software tools.

**FIG 1:**
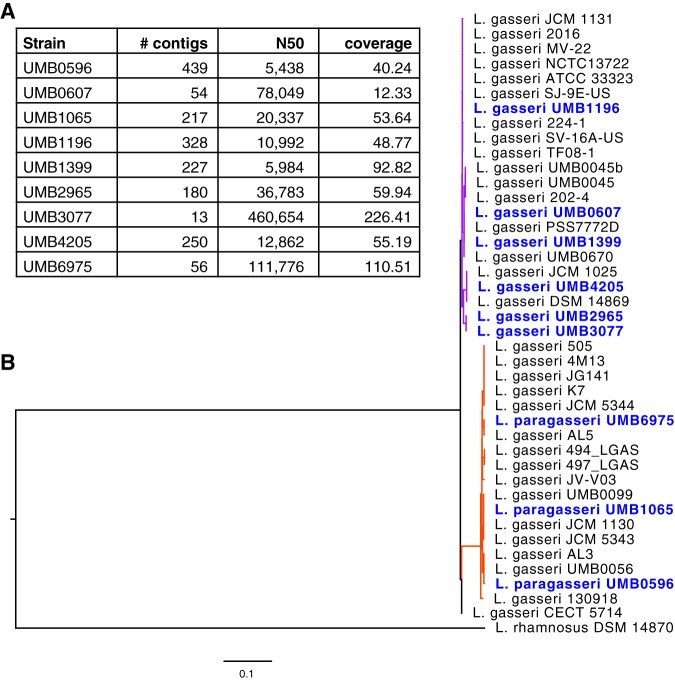
Genome assembly statistics (A) and phylogenetic tree (B) of six L. gasseri and three *L. paragasseri* strains. Genomes sequenced in this study are shown in blue. Branches belonging to the L. gasseri clade are shown in purple, and the *L. paragasseri* clade’s branches are shown in orange.

The nine bladder lactobacilli genomes vary in size from 1,041,937 bp (strain UMB1399) to 2,108,391 bp (strain UMB6975) in length, with an average GC content of 35.2%. Assembly statistics are listed in Fig. 1A. Genome assemblies for lactobacilli are particularly challenging given the presence of numerous short repeats throughout the genome (14). As part of NCBI’s quality control process, average nucleotide identity is calculated (15), and three of the genomes (UMB0596, UMB1065, and UMB6975) were reclassified as strains of the species *L. paragasseri* (98 to 99% identical for over 94% of the genome to the type genome of *L. paragasseri* (strain JCM 5343 [GenBank accession number AP018549]). The nine bladder lactobacillus genomes were also compared with those of publicly available L. gasseri and *L. paragasseri* strains in PATRIC (as of July 2019). Figure 1B shows a phylogenetic analysis of these genome comparisons. There is a clear distinction between the six bladder L. gasseri and three bladder *L. paragasseri* strains. From our phylogenetic analysis, we have identified not only three new strains of *L. paragasseri* but also other strains presently classified as L. gasseri that are likely members of the *L. paragasseri* species (Fig. 1B; branches shown in orange).

### Data availability.

This whole-genome shotgun project has been deposited in GenBank under the accession numbers VNFS00000000 (UMB4205), VNFT00000000 (UMB2965), VNFU00000000 (UMB1196), VNFY00000000 (UMB0607), VNGC00000000 (UMB3077), and VNGD00000000 (UMB1399) for the six L. gasseri strains and VNFQ00000000 (UMB0596), VNFV00000000 (UMB1065), and VNFX00000000 (UMB6975) for the three L. paragasseri strains. The versions described in this paper are the first versions. Raw sequence data are publicly available for the six L. gasseri strains (SRA accession numbers SRR9695707, SRR9695712, SRR9695713, SRR9695714, SRR9695719, and SRR9695724) and the three *L. paragasseri* strains (accession numbers SRR9695720, SRR9695721, and SRR9695723).
